# “I Am Reluctant to Continue, Yet Know She Could Go into Withdrawal”: A Qualitative Analysis of Clinician Requests for eConsults in Opioid Management in the Ambulatory Setting

**DOI:** 10.1007/s11606-025-09811-0

**Published:** 2025-09-02

**Authors:** Laila Khalid, Sharon Rikin, Dana Watnick, Tiffany Y. Lu, Gianni Carrozzi, Ana Valle, Joanna L. Starrels

**Affiliations:** 1 Montefiore Medical Center, Bronx, NY USA; 2 Albert Einstein College of Medicine, Bronx, NY USA; 3 New York City Health and Hospitals/Jacobi, Bronx, NY USA; 4 Brigham and Women’s Hospital, Boston, MA USA

**Keywords:** eConsults, opioid management, qualitative analysis, outpatient

## Abstract

**Background:**

Electronic consultations (eConsults) allow clinicians to submit questions through the electronic health records (EHR) to request specialist input about individual patients’ care, but there is paucity of literature describing eConsult programs specifically for opioid management.

**Objectives:**

1. To understand clinicians’ reasons for seeking eConsults for opioid management.

2. To describe characteristics of clinicians’ questions that can be addressed through eConsults without requiring in-person specialist visits.

**Design:**

Retrospective qualitative study of eConsults for opioid management in the ambulatory setting.

**Participants:**

Clinicians who submitted an eConsult request for opioid management.

**Approach:**

We conducted a qualitative content analysis of eConsult requests and responses. First, a priori codes were developed and applied to textual data. Through iterative discussion and memoing, thematic codes were identified and applied to the dataset. Subsequently, breadth and depth of codes were explored, collapsed, and redefined, and relationships between codes were discussed. Following theme development, matrix analysis was used to compare eConsults that resulted with or without a recommendation for an in-person specialist visit.

**Key Results:**

Forty eConsults were requested by 30 unique referring clinicians mostly from general internal medicine (53%) or family medicine (27%). We identified four themes: (1) clinicians were motivated to reduce harm, (2) clinicians had difficulty labeling opioid misuse and use disorder (OUD), (3) clinicians had difficulty articulating discrete questions, and (4) clinician questions revealed knowledge gaps in opioid management. In-person specialist visits were recommended when clinicians needed help with labeling opioid misuse or OUD or when questions were vague and around complex topics.

**Conclusion:**

Clinicians have unease and discomfort in treating chronic pain and managing opioids. eConsults are most helpful for low-complexity, discrete questions from clinicians focused on reducing harm. eConsult implementation should integrate EHR enhancements and clinician training in assessing opioid misuse and OUD and articulating discrete questions.

## INTRODUCTION

While opioid prescribing for chronic pain has substantial risks and is not supported by evidence of efficacy,^1^ in 2021, nearly a quarter of adults in the USA with chronic pain used a prescription opioid in the last 3 months.^2^ The largest group of providers prescribing opioids for chronic pain are primary care providers (PCPs), who prescribed approximately 37% of all opioid prescriptions.^3^ Many PCPs and other clinicians find opioid management challenging due to lack of training and insufficient access to specialists in pain and addiction.^3,4^ Thus, there exists a strong need to support clinicians in opioid management.

One model for addressing this need is electronic consultation (eConsult) services. eConsults offer a platform for clinicians to request specialist input in the care of individual patients in an efficient manner, within the electronic health record (EHR),^5^ reducing the need for in-person specialist visits.^6^ While technologies and models are evolving, we consider an eConsult to be characterized by a question initiated by a referring clinician about an individual patient that is electronically submitted to a physician specialist team, who respond electronically to the referring clinician. In the USA and at our institution, ambulatory eConsults have been implemented for a range of medical and surgical sub-specialties such as hematology, endocrinology, rheumatology, and urology.^7–9^

We describe an eConsult service that was established by general internal medicine physicians with expertise in long-term opioid therapy for chronic pain to provide guidance around opioid management in the ambulatory setting. While models for providing electronic guidance on pain and opioid management have been described, including in the Veterans Affairs (VA) health system, the eConsult service described here differs from prior models reported in the literature in that prior interventions have not been driven exclusively by clinician-initiated questions electronically submitted to a physician specialist team who then respond electronically to the referring clinician.^10,11^ Prior published eConsult interventions have also focused on pain or addiction or urine drug testing, but few have been specifically developed for opioid management. Further, while we are aware of one qualitative study of pain management eConsults in Canada, prior qualitative studies have not examined eConsults for opioid management.^12^ To our knowledge, there are no prior qualitative studies describing a clinician-initiated eConsult model specifically for opioid management.

In this qualitative study, we examined the content of eConsults to identify themes and explore thematic differences in eConsults that resulted with or without a recommendation for an in-person specialist visit.

## METHODS

### Setting and Study Design

This study was conducted at Montefiore Medical Center (Montefiore), a large academic medical center in the Bronx, NY. Montefiore serves primarily Black and Hispanic patients and nearly 80% are publicly insured with Medicare and/or Medicaid. Between 2019 and 2020, a mean of 1721 patients per month were prescribed long-term opioid therapy. Ambulatory pain management specialists at Montefiore do not prescribe long-term opioid therapy, which are managed by PCPs and some specialists.

We conducted a combined deductive and inductive content analysis of the text from eConsults for opioid management including (1) questions by referring clinicians and (2) recommendations by eConsultants, from July 1, 2019 to September 30, 2020. The study was approved by the institutional review board of Montefiore and Albert Einstein College of Medicine.

### Description of Opioid Management eConsults

At Montefiore, ambulatory eConsults have been available for medical sub-specialties such as cardiology, hematology, and endocrinology since 2018. When eConsults are ordered, clinicians are prompted to ask the eConsultants specific questions about the patient, and eConsultants respond asynchronously within the patient’s EHR. These have been described previously.^9^

In 2019, the eConsult for opioid management became available within the platform. Three general internal medicine physicians (TL, JLS, LK), who were boarded in addiction medicine with expertise in opioid management and saw patients with chronic pain at their respective ambulatory clinic sites were recruited as eConsultants to review and respond to eConsults. eConsults for opioid management were made available to all Montefiore ambulatory settings, including primary care and specialty clinics. Through e-mails and announcements at meetings, clinicians were made aware of the eConsults for opioid management which were intended “for questions about [opioid] dosage, tapering, drug screen interpretation, and opioid use disorder.” Clinicians requesting an eConsult responded to two prompts: (1) *Please provide a brief summary of the patient*, and (2) *Please provide your questions for the eConsultant*. Within 72 h, one of the eConsultants reviewed the eConsult question and responded with their recommendations. eConsultants completing eConsults received guidance that eConsults should be completed in less than 15 min. When addressing clinical questions, eConsultants used a common definition for opioid misuse (i.e., use in a way other than as prescribed) and OUD (per Diagnostic Statistical Manual, Fifth Edition).^13^ eConsultants also considered and indicated in the response whether a specialist appointment was recommended to fully address the clinician questions. Incentives for referring clinicians ($17.50) and eConsultants ($30) were given for each eConsult completed to offset time spent for the service, similar to other eConsults. During the study period, the referring clinicians’ and eConsultants’ remuneration was supported by the institution.

### Data Collection and Management

We included data for every eConsult during the study timeframe. Each unique patient had only one eConsult; however, a clinician could have requested more than one eConsult for different patients. For each eConsult, a systematic chart review and abstraction with double data-entry was performed by two members of the research team (an internal medicine resident physician [AV] and a specialist [LK]). We abstracted demographic characteristics (age, sex, race/ethnicity), full text of the eConsult (clinician question and eConsultant recommendation); the date of eConsult; and referring clinician specialty. Textual data from the eConsult were uploaded to and analyzed using the Dedoose platform (9.0.85, Los Angeles, CA).^14^

### Data Analysis

Data were analyzed by addiction medicine and pain specialists (LK, JLS), a quality improvement researcher (SR), and a qualitative research expert (DW). The unit of analysis was the patient, with textual data from the eConsult. First, the lead author (LK) developed a priori codes based on clinical experience, existing theory, and academic literature. At weekly meetings, the analytic team discussed codebook development, including identifying repeating ideas, defining codes, and questioning negative cases to refine codebook application.^15^ Two authors (LK, AV) applied the codebook to the full dataset, discussed discrepancies, and reported back to the full group for iterative feedback, revision, and reapplication until thematic saturation was adequately achieved.^16^ Concurrent to codebook development, thematic and case memos were written to capture emergent themes and relationships between codes. Later in theme development, we incorporated personal clinical reflections to support reflexive review of our analytic assumptions, and compared our findings to existing literature. In ad hoc analysis, we then used matrices to review how overall themes manifested among cases that were either sufficiently resolved with an eConsult vs. those which the responding eConsultant determined to need further consultation through an in-person specialist visit.

## RESULTS

Forty eConsults were requested by 30 unique referring clinicians, 28 physicians (93%) and 2 nurse practitioners (7%). Referring clinicians practiced general internal medicine (53%), family medicine (27%), hematology (7%) gynecology/obstetrics (10%), and geriatric medicine (3%). Both nurse practitioners were from the hematology service. Most patients were female (65%), black (60%), and publicly insured (77.5%), with a mean age of 53 years (range 27–86 years). Seventy-five percent of the patients were prescribed opioids at the time of the eConsult (the remainder were prescribed opioids elsewhere and/or intermittently, were transitioning care, and/or the questions were about substances other than prescribed opioids). eConsultants responded to all 40 eConsults and recommended an in-person visit with a specialist in 15 of the 40 (37.5%) requests.

We identified four themes: (1) clinicians were motivated to reduce harm, (2) clinicians had difficulty labeling opioid misuse and OUD, (3) clinicians had difficulty articulating discrete questions around opioid management, and (4) clinician questions revealed knowledge gaps in opioid management. Here, we present four themes, then describe differences in themes between eConsults that resulted with or without a recommendation for an in-person specialist visit.

### Theme 1: Clinicians Were Motivated to Reduce Harm

eConsult questions consistently revealed that clinicians’ underlying motivation was to reduce harm to their patients, which included balancing the harms of ongoing chronic pain with the harms of prescribing, and of not prescribing opioids.

Clinician questions revealed their desire to reduce persistent pain. One asked for help on “*how to manage this, as she feels like* [her pain is] *not being managed effectively at all.*” Another clinician requested help in managing opioids because of poor pain control: “*Given recurrent pain and hospitalizations, failed use of [pregabalin], and not tolerating Q8H of narcotic PRN, should the narcotic PRN frequency be increased?*”.

Clinicians also expressed intent to reduce harm to patients through judicious opioid prescribing, i.e., avoiding prescribing high doses of opioids, limiting refills of opioid prescriptions, and by tapering or stopping opioids. One clinician expressed concern that non-prescribed medications present in the urine drug screen could increase the risk of overdose in a patient on prescribed opioids: “*Utox has been positive for non-prescribed medications*,” concluding, “*therefore risks of medication will not outweigh benefits.*” Another clinician requested help for a patient that they were covering for another clinician. The patient was prescribed opioids but was not following through with multimodal specialist appointments and the clinician was unable to assess the patient in person due to COVID-19 precautions. The unease with not being able to fully assess benefits and harms of prescribing opioids while also understanding consequences of withholding opioids was expressed by, “*I am reluctant to continue the refill cycle and yet know that she could go into withdrawal…*” By acknowledging the other side of the risk equation (in this case, that not prescribing opioids also has risks, e.g., of withdrawal), this quote exemplifies providers’ balancing of risks.

Clinician questions about how to reduce harm ranged from seeking reassurance from the specialist about a management plan to leaving opioid prescribing decisions entirely to the specialist. For example, one clinician identified a patient as being a “*candidate for buprenorphine*” and sought eConsultant’s reassurance in switching the patient to buprenorphine. Other clinicians preferred to hand off management: “*Would like a pain specialist recommendation or appointment for evaluation.*”

### Theme 2: Clinicians Had Difficulty Labeling Opioid Misuse and OUD

Clinician difficulty labeling opioid misuse and OUD was evident by examples of either not labeling misuse or OUD when they seemed likely from the details provided. Conversely, clinicians also sometimes assumed misuse or OUD when they did not seem likely based on details provided.

Clinicians’ avoided explicitly labeling misuse or OUD. For example, one clinician described use of prescribed opioids more frequently than prescribed, without naming misuse: “*…on chronic high dose opioids and reports she has been running out of pain meds early.*” Another clinician wrote that a patient “*was being prescribed [oxycodone/acetaminophen] in the past but stated she was eating them like candy.*” Similarly, another clinician mentioned use of non-prescribed controlled substances, without identifying it as misuse: “*recent urine tox screen positive for benzos (obtained from friend for anxiety), … also positive for oxycodone. Patient denies using [oxycodone/acetaminophen], [long-acting oxycodone], *etc.*….*”

Clinicians also prematurely concluded that there was opioid misuse or assumed higher severity of opioid misuse than was described in the patient’s chart. For example, one clinician considered cannabis use as a potential sign of diversion of prescribed opioids, writing that the patient “*has been prescribed chronic opioid therapy ….by previous provider who has left …. Utox has been positive for non-prescribed medications [cannabis]. Concern that based on these results there is a risk of diversion.*”

Clinicians had already made management decisions without clarifying if these were supported by full assessments of misuse and OUD. One clinician decided to discontinue long-term opioids for a patient with one diluted urine drug test, presuming that urine tampering occurred even though the patient was taking a diuretic, “*tested urine for drug screening and found to be negative…* [for] *opioids likely due to dilution (low Cr)….. At this time I am not continuing narcotics for treatment of pain.*”

### Theme 3: Clinicians Had Difficulty Articulating Discrete Questions around Opioid Management

Clinician requests revealed that although eConsults were clinician-initiated and referring clinicians were prompted to ask the eConsultant a specific question, the questions submitted were often not specific or missing information required by the eConsultant to adequately respond.

Clinicians prominently expressed discomfort without asking a specific question, reflecting general unease around pain or opioid management. For example, one clinician wrote: “*On oxycodone 30 mg 210 tabs a month [for sickle cell pain]; will not send this much narcotics…. and would require pain specialist recommendation.*” Although the clinician wrote that they would not continue a patient’s high-dose opioid regimen, they did not ask the specialist a question around opioids. They also did not describe their decision-making or provide additional information to guide next steps.

Another clinician was considering tapering opioids for a patient who had been using a low and stable dose without providing information about their assessment of the risks or benefits for that individual, stating “*current regimen [is] gabapentin 900 mg TID, tramadol 50 mg Q6H. Also on [citalopram] and buspirone for anxiety/depression…. [Recommendations requested for] tapering of chronic opioid with control of chronic lumbar radiculopathy.*”

Clinicians did not provide key information required by the specialist to provide guidance. For example, one clinician indicated that the patient was receiving methadone without specifying whether it was for chronic pain or OUD and did not ask a question, stating that the patient was “*on methadone chronic [from] outside clinic,* … *requesting opioid medication for pain….I have given tramadol.*” Methadone can be prescribed by ambulatory clinicians for treatment of chronic pain, or it can be dispensed at opioid treatment programs for treatment of OUD. Differentiating between etiology of use is important in considering a patient’s management plan but this information was not included in the eConsult.

### Theme 4: Clinician Questions Revealed Knowledge Gaps in Opioid Management

eConsults revealed knowledge gaps among clinicians on specific topics in opioid management practices, such as pharmacology of opioids, guideline implementation, as well as use of eConsults in the pertinent clinical setting.

Clinicians requested guidance to switch patients from short-acting to long-acting opioid medications to improve pain control, a historical practice that has not been supported by evidence. One clinician wrote, “*Could you please assist in suggesting longer acting formulations, the [oxycodone/acetaminophen] does control her pain [only] for the three hours at this dose.*”

Another clinician had misconceptions about guidelines for opioid prescribing for chronic pain, believing that it is necessary to observe urine collection: “*We are unable to observe her urinating and validate that she is proving a valid urine sample and have concerns… therefore [we are] unable to follow safe prescribing guidelines.*”

Although eConsults for opioid management were developed for use in ambulatory settings and specifically for opioid management, some clinicians used the eConsults for hospitalized patients or for guidance on non-opioid substances. One clinician wrote: “*[Patient is] now postop and interested in returning to [buprenorphine/naloxone]… What would you recommend for timing of [buprenorphine/naloxone] given she is postop?*” Another clinician wrote about an admitted patient “… *41wks pregnant in early labor p/w sx of opioid withdrawal and with COWS score of 13. Pt currently on [buprenorphine/naloxone] 4 mg in am and 2 mg in PM with last dose last night. Also with amphetamine use…. Help managing likely moderate withdrawal.*” A clinician seeking help for a patient’s cocaine and cannabis use wrote: “*Hx of Cocaine, Marijuana use ….Needs Detox and long term Drug Rehab.*” These examples highlight the desire for specialty consultation for pain and addiction in not only the ambulatory setting, but also in the inpatient setting.

### Results of Matrix Analyses: The Four Themes Influenced Whether an In-Person Visit with a Specialist Was Recommended

Differences in themes were noted between eConsults that resulted with or without a recommendation for an in-person specialist visit. In-person specialist visits were generally recommended when: clinicians did not articulate specific harms they were concerned about (theme 1), were not explicit about misuse or OUD and had not labeled it as such (theme 2), their question was vague or indicated a high-complexity plan that was not justified (theme 3), or clinician knowledge gaps seemed to surpass what could be addressed in an eConsult (theme 4). In-person specialist visits were not perceived to be necessary when the clinician articulated specific questions, provided the information the specialist needed, and were low complexity without involving complex queries on opioid tapering, switching opioids, or overall pain management. Table 1 presents the matrix analysis of themes by whether a visit was recommended.

**Table 1:** eConsult Themes and eConsultant Recommendations for In-Person Visits

Theme	Examples of how themes influenced visit outcome	
	Yes, in-person specialist visit was recommended when:	No, in-person specialist visit was not needed when:
1. Clinician motivated to reduce harm	• Clinicians did not articulate potential or observed harms	• Clinician could articulate patient harms
2. Clinician had difficulty labeling misuse and OUD	• Clinician mentioned concerning behavior but there was no diagnosis or further assessment of misuse or OUD	• Clinician asked a discrete question about assessing misuse (e.g., help with urine drug screen interpretation)
3. Clinician had difficulty articulating discrete questions	• No clear question about a high-complexity plan (e.g., tapering off high-dose opioids or switching to buprenorphine) • General request for an entire pain management plan	• Questions were clearly stated • Ambiguity in questions was about low-complexity issues
4. Clinician questions revealed knowledge gaps in opioid management	• General knowledge gap raised concern that clinician would not be able to follow through with recommendations • Clinician suggested a plan that could cause harm	• Discrete knowledge gap amenable to teaching within an eConsult • Incorrect use of eConsult (e.g., for other substances or during an inpatient admission)

## DISCUSSION

This study described use of an innovative model of care, eConsults, for opioid management for patients with chronic pain. Qualitative analysis revealed that eConsults can provide guidance for discrete questions and a next step for clinicians when they feel inadequately prepared to navigate opioid prescribing and risks, but eConsults may not be sufficient to fully provide a pathway forward for complex cases and questions. In this study of eConsults for opioid management, we found that the majority of eConsults were addressable without need for an in-person specialist visit. However, a specialist visit was still necessary for nearly 40% of the requests, particularly when the specialist perceived clinical urgency or if there was inadequate information and assessment provided by the clinician. eConsults were most efficient at guiding care for low-complexity, discrete questions around a specific teachable topic and did not involve more complex queries on opioid tapering, switching opioids, or overall pain management, which require a more nuanced response and likely and in-person specialist visit.

We found that clinicians were motivated to reduce harms of opioid prescribing, but they had difficulty labeling misuse or OUD or articulating their questions. We believe that underlying these difficulties is a deeper, perhaps unrecognized, discomfort or unease in treating chronic pain and managing opioids. Prior studies have demonstrated clinician discomfort in managing chronic pain and weighing risks and benefits of opioid prescribing.^17–20^ Clinicians value patient safety and want to avoid contributing to harms such as overdose or addiction, resulting in caution when considering or prescribing opioids.^21–23^ More recent attention to harms of discontinuing or reducing prescribed opioid dose further complicate opioid prescribing decisions.^24,25^ Support or validation from specialists can help clinicians be more confident in decision-making and engaging patients in conversations around opioid prescribing.^12,22,26,27^ eConsults for opioid management is a feasible way for clinicians to seek timely support from specialists and for patients to benefit from specialist perspectives. eConsults also helped clinicians identify patients who required a specialist visit.

Although concern for opioid-related harms was high, this study also revealed that referring clinicians often lacked the language and were unable to accurately describe their concerns about patients’ opioid misuse or OUD and label it as such. Labeling misuse or OUD is the first step prior to subsequent clinical decision-making. Guidelines recommend that clinicians who treat chronic pain should be competent in assessing and addressing OUD.^3^ However, there is a well-documented gap in knowledge and training in treatment of chronic pain, opioid management, and addiction.^4,22,28–30^ Clinician difficulty assessing for OUD in individuals taking long-term opioid therapy for chronic pain is particularly challenging, since criteria for tolerance, withdrawal, and long duration of use do not readily apply. Our findings suggest that difficulty labeling misuse and OUD is unlikely to be overcome with an eConsult alone and requires an in-person specialist visit. Other initiatives to enhance clinician knowledge are needed, such as training, mentorship, case-based presentations, or videoconferencing with specialists.^31–35^

In considering efficiency of the eConsults, i.e., whether in-person specialist visits were recommended, we found that in 37.5% of the eConsults, a specialist visit was recommended, similar to eConsults for other specialties at our institution as well as elsewhere.^36–38^ In general, eConsults in opioid management that could be addressed without a specialist visit asked discrete questions about low-complexity patient situations. Similar findings were observed in a study by our team which found that rheumatology eConsults resulted in a recommendation for an in-person specialist visit when there was a need for a physical examination and eliciting information directly from patients.^39^ In addition, we found that some questions to eConsults were not appropriate for the eConsult service because they were for inpatients or about substances other than opioids. Based on these findings and clinical experience, we believe that future opioid management eConsult initiatives should incorporate EHR enhancements including templates that use checkboxes or other specific prompts and structured fields to promote efficient eConsults. These can include providing prompts to describe and assess behaviors reflecting misuse and using DSM-5 criteria to diagnose OUD and prompts to indicate patient-specific risks and benefits of opioids. In addition to enhancing the eConsult, improvements could also include educational interventions, mentoring by specialists, and development of collaborative multidisciplinary models like the VA.^34,40–42^ eConsults or telementoring of clinicians not only helps increase communication with specialists but can also change opioid prescribing practices.^10,35,43^

There are several limitations to this study. The sample was 40 eConsults from a single healthcare system during a brief period of time, and findings may not be generalizable to other contexts. Regional differences, secular trends, and patient population can impact opioid prescribing practices, clinician questions, and the proportion of eConsults for which in-person visits are recommended. In this healthcare system, use of the eConsult for opioid management was low, and we are unable to determine why more clinicians prescribing opioids did not use the eConsult to request input for more patients. We also could not determine the time spent by eConsultants in answering eConsults. eConsultant decisions about when to recommend an in-person visit may have been influenced by other factors not represented in the eConsult data, such as availability of appointments. Many eConsult questions were short in length, and it is possible that emerging themes would have been different if additional information had been provided. Because we analyzed existing text, it was not possible to explore intent or other considerations not included in the text of the eConsult, or clinicians’ motivation and ability to implement eConsultant’s recommendations; future studies can examine how clinicians interpret and respond to opioid management eConsult recommendations and to what extent these recommendations change practice.

Overall, we found that clinicians’ difficulty labeling misuse and OUD and navigating the risks of opioid prescribing is a reason to offer an eConsult model for opioid management, but may also reduce its efficiency. Thus, eConsults do not obviate the need for in-person specialist assessments. These findings can inform future implementation of eConsults for opioid management, which could benefit from clinician training and EHR enhancements.
